# Calreticulin—Multifunctional Chaperone in Immunogenic Cell Death: Potential Significance as a Prognostic Biomarker in Ovarian Cancer Patients

**DOI:** 10.3390/cells10010130

**Published:** 2021-01-11

**Authors:** Michal Kielbik, Izabela Szulc-Kielbik, Magdalena Klink

**Affiliations:** Institute of Medical Biology, Polish Academy of Sciences, 106 Lodowa Str., 93-232 Lodz, Poland; iszulc@cbm.pan.pl (I.S.-K.); mklink@cbm.pan.pl (M.K.)

**Keywords:** calreticulin, immunogenic cell death, ovarian cancer, DAMPs, chaperones

## Abstract

Immunogenic cell death (ICD) is a type of death, which has the hallmarks of necroptosis and apoptosis, and is best characterized in malignant diseases. Chemotherapeutics, radiotherapy and photodynamic therapy induce intracellular stress response pathways in tumor cells, leading to a secretion of various factors belonging to a family of damage-associated molecular patterns molecules, capable of inducing the adaptive immune response. One of them is calreticulin (CRT), an endoplasmic reticulum-associated chaperone. Its presence on the surface of dying tumor cells serves as an “eat me” signal for antigen presenting cells (APC). Engulfment of tumor cells by APCs results in the presentation of tumor’s antigens to cytotoxic T-cells and production of cytokines/chemokines, which activate immune cells responsible for tumor cells killing. Thus, the development of ICD and the expression of CRT can help standard therapy to eradicate tumor cells. Here, we review the physiological functions of CRT and its involvement in the ICD appearance in malignant disease. Moreover, we also focus on the ability of various anti-cancer drugs to induce expression of surface CRT on ovarian cancer cells. The second aim of this work is to discuss and summarize the prognostic/predictive value of CRT in ovarian cancer patients.

## 1. Introduction

Cell death is an important process that plays a great role in the development and maintaining of the homeostasis of every living organism. There are many different types of eukaryotic cell death, classified depending on morphological changes inside the cell (i.e., shrinkage of the cytoplasm, chromatin condensation, nuclear fragmentation, degradation of intracellular organelles, rupture of cell membrane); enzymological criteria (i.e., participation or not of nucleases or proteases); functional aspects (i.e., programed, accidental, physiological, pathological); immunological character (immunogenic or non-immunogenic). Liu et al. [1,2,3] performed literature research and counted that there are over 34 different cell death modes, among which best-known and most studied are apoptosis, necrosis, autophagy, necroptosis, anoikis and pyroptosis. The traditional process of apoptosis is a non-immunogenic one, even termed as tolerogenic cell death, that actively inhibits immune reaction [4,5]. In contrast, necrosis is uncontrolled, immunologically harmful process in which increased secretion of pro-inflammatory cytokines as well as recruitment of different immune cells leading to the development of immune responses in the tissue are observed [1,6,7,8]. Necroptosis (programed necrosis) is a regulated cell death type that mimics features of both apoptosis and necrosis and is also known to induce immune-related processes [9,10]. Apoptosis, on the other hand, under certain conditions, may occur in the way that the immune system is alerted, triggering immunity against the dying cell, which releases its cellular content into the microenvironment, that in turn leads to the recruitment and activation of immune cells. Such a form of apoptosis, which may also occur in the context of necroptosis, is called immunogenic cell death (ICD) [11]. ICD is characterized by the emission of particular molecules that induce the immune response. This kind of cell death is especially important and best characterized in malignant diseases. One of the best-known/most important molecules implicated in ICD is chaperone called calreticulin [12,13].

Calreticulin (also termed as CRT or CALR) is an endoplasmic reticulum (ER)-associated chaperone with numerous biological activities. Its functions strongly depend on cellular localization. In ER it is a main regulator of Ca^2+^ homeostasis and is also responsible for loading of cellular antigens into major histocompatibility complex (MHC) class I molecules. In cytoplasm, calreticulin is considered to be integrin activator and mediator of integrin-dependent cell adhesion [14,15,16]. Very important biological role of CRT is connected with cell death. In stressed or dying cells this chaperone is expressed on the surface of cell membrane or even release into extracellular milieu [17]. Plasma membrane CRT (also called ecto-CRT) serves as a co-stimulatory and pre-mortem (“eat-me”) signals to variety of immune cells but mainly to antigen presenting cells (APCs). Moreover, ecto-CRT is one of the main hallmarks of ICD in malignant diseases. Calreticulin present on outer membrane of tumor cells not only stimulates their phagocytosis by APCs, but also activates the adaptive immune response, thus it is believed to be crucial for surveillance against tumors [18,19,20]. The role of CRT in ICD in malignant diseases have been recently widely studied on both in vitro (cell lines) and in vivo (animals, patient’s tumor specimens) models. However, up to this date, available data is still scarce and highly controversial. As it has been shown in published research, ecto-CRT may be either positive or negative marker of patients/tumor cells survival, depending on type of tumor [21,22].

One of the most dangerous malignant diseases is ovarian cancer. The mortality of patients suffering from this kind of tumor is still very high in both the United States and Europe. The 5-year-survival of women with advanced ovarian cancer is very low and does not exceed 25%. There are a number of reasons for such a poor outcome. Among the most important are: late diagnosis of the disease (an overwhelming number of patients are diagnosed in the advanced stage of disease), high resistance of cancer cells to chemotherapeutics, unique metastatic properties and poor immunogenicity of ovarian cancer cells [23,24,25]. It is considered that better identification and evaluation of immune-modulating therapeutic approaches can be one of the pathways to overcome poor prognosis of ovarian cancer patients. In this context ICD might be a promising way [24].

This review is dedicated to summarize the role of CRT in keeping the homeostasis of normal cell’s, as well as in the development of ICD in malignant diseases. Moreover, the focus on CRT as a prognostic factor in ovarian cancer patients and ability of chemotherapeutics to induce ecto-CRT in ovarian cancer cells is also the goal of this paper.

This is narrative literature review. PubMed library was the main data source and search strategy was based on keywords (i.e., chaperones, calreticulin, immunogenic cell death, cell death, ovarian cancer, prognostic factor, chemotherapeutic, platinum compounds, anthracyclines, gemcitabine, taxanes) used separately or in combination to cover assumed chapters. The review includes only peer-reviewed articles and journals with one exception—the guideline for ovarian cancer patients’ treatment available online. The conference abstracts or letters to the Editor were excluded. The period of this review covers the years from 1990 to November 2020. The data concerning CRT in ovarian cancer was collected from 2010 to November 2020.

## 2. Immunogenic Cell Death—ER Stress Connection

ICD has been defined as an unique class of regulated cell death that is able to elicit complete antigen-specific adaptive immune response through the emission of particular molecules that belong to a class of the damage-associated molecular patterns (DAMPs) family [12,26]. ICD is a stressor-dependent cell death, since it is induced as an effect of ER stress combined with the generation of reactive oxygen species (ROS). In response to stress conditions, when there is accumulation of misfolded proteins, ER initiates the activation of a complex signaling pathways network, called the unfolded protein response (UPR). There are three different ER membrane-associated sensors that initiate UPR signaling pathway: protein kinase R-like endoplasmic reticulum kinase (PERK), activating transcription factor-6 (ATF6) and inositol-requiring transmembrane kinase/endoribonuclease 1 (IRE1) [27,28]. PERK is one of the major signaling pathways responsible for attenuation of mRNA translation under ER stress, preventing the influx of newly synthesized proteins into ER compartments. This type I transmembrane protein, when activated, triggers the phosphorylation of *α*-subunit of eukaryotic initiation factor 2 (eIF2*α*), leading to the inhibition of polypeptide chain synthesis. Generally, the activity of PERK pathway may result in cell cycle arrest, and under prolonged or severe stress conditions activation of this kinase enhances the apoptosis-signaling pathway [27,29]. ATF6, a type II transmembrane protein located on the ER surface, is a basic leucine zipper transcription factor which, after exposure to stressful conditions, translocates to the Golgi complex. Over there it is cleaved by site-1 and site-2 proteases and generated fragment then translocates to the nucleus where it can up-regulate the expression of genes encoding proteins involved in the UPR, including ER chaperones and transcription factor X-box-binding protein 1 (XBP1). IRE1, a type I transmembrane protein, after oligomerization and autophosphorylation, activates XBP1, which further induces the expression of UPR stress genes [27,28,30]. During prolonged stress exposure, IRE1 was also considered to be a key initiator of ER stress-induced cell death, as it interacts with tumor necrosis factor receptor associated factor 2 (TRAF2). This leads to the activation of apoptotic-signaling kinase-1 (ASK1), which induces downstream kinases: Jun-N-terminal kinase (JNK) and p38 mitogen activated protein kinase (MAPK), and in consequence promote apoptosis [29,31].

ICD has been well characterized in the context of tumor therapy, as its induction is one of the mechanisms, by which conventional chemotherapeutics can kill tumor cells. However, there is a range of specific stimuli that can initiate ICD. Among them are: (a) obligatory intracellular pathogens, including numerous bacterial and viral species; (b) conventional chemotherapeutics, such as anthracyclines, some DNA-damaging agents, proteasomal inhibitors, poly-A-ribose polymerase inhibitors or mitotic poisons; (c) anti-cancer agents that target different types of cell components or processes inducing cell death pathways; (d) various oncolytic potential molecules, i.e., viruses; (e) some chemicals; (f) physical stressors, such as ionizing radiation, hypericin-based photodynamic therapy, high hydrostatic pressure, nanopulse stimulation or severe cytotoxic heat shock [19,32,33].

Generally, based on their different mechanisms of action involved in ER stress, ICD inducers are divided either into type I or type II. Type I inducers initiate ER stress indirectly, as a downstream effect, while type II inducers can directly launch the ER stress response, provoking cell death [26,34]. Among the type I inducers are the best-known anti-cancer drugs, such as anthracyclines (doxorubicin, mitoxantrone), taxanes (paclitaxel, docetaxel), gemcitabine, cyclophosphamide, fluorouracil (5FU), and alkylating agent melphalan. Platinum derivatives such as cisplatin and carboplatin are believed to be controversial and their effectiveness to resolve ICD depends on the type of tumor cells, concentration used and treatment duration. In contrast, oxaliplatin, a third-generation platinum analogue, seems to be indisputable type I ICD inducer. On the other hand, physical methods, such as photodynamic therapy, high hydrostatic pressure or molecules as oncolytic viruses are the example of type II inducers [30,35,36,37]. Both types of inducers, by the combined action of ER stress and ROS generation which activates danger signaling pathways, are able to trigger the timely release or membrane exposure of a series of DAMPs. These endogenous danger molecules interact with a range of scavenger receptors (i.e., LDL receptor-related protein, LRP1/CD91), purinergic receptors (i.e., P_2_RX_7_/P_2_RY_2_) and pattern recognition receptors (PRR, i.e., Toll-like receptor 4—TLR4) on the innate immune cells, such as monocytes, neutrophils, macrophages or dendritic cells (DCs) [38].

Since the introduction of the danger model in 1994 [39], numerous DAMPs have been identified, and still the new ones are characterized. DAMPs are molecules that can be released, both, from extracellular matrix and intracellular compartments (ER, nucleus, cytosol, plasma membrane, mitochondria) as the response of dying cell [40]. The overall role of ICD-associated DAMPs involves: facilitating the recruitment of APCs or their precursors to the site of ICD; guiding the interaction between dying cell and APCs; triggering the phagocytosis of dead cells or their leftovers; promoting the maturation of APCs and their ability for effective antigen cross-presentation or enabling the recruitment of T-cells [19]. Among DAMPs most frequently associated with ICD are CRT, adenosine-5′-triphosphate (ATP), non-histone chromatin-binding protein—high-mobility group box 1 (HMGB1), type I and II interferons (IFN), annexin A1 and heat shock proteins (HSPs) 70 and 90. The majority of these molecules have non-immunogenic functions inside cell, but when exposed on cell surface or released extracellularly they become immunogenic [22,35,36].

Calreticulin will be thoroughly described in the next chapter of this manuscript. ATP, under physiological conditions, is involved in various cellular metabolic processes and intracellular responses, but during apoptosis its releasement from dying cell occurs in autophagy-dependent manner. The presence of ATP in extracellular space acts as “find me” signal, being a chemoattractant for DCs precursors. ATP binds to the P_2_RX_7_ receptor on DCs, providing the inflammasome-mediated secretion of interleukin 1β (IL-1β), an important cytokine that plays crucial role in immune response development [41]. Another DAMP triggered by ICD is HMGB1, normally found in the nucleus (but with cytoplasmic localization due to shuttling), that serves various nuclear and cytosolic functions. HMGB1 is released from dying or stressed cells and when present in the extracellular space, can signal tissue injury. Through binding to a range of receptors, including TLR2, TLR4 and the receptor for advanced glycosylation end products (RAGE), this molecule can initiate the inflammatory response. However, it should be stressed that TLR4 seems to be the exclusive HMGB1 receptor and it is relevant in the context of considering cell death as immunogenic. HMGB1-TLR4 triggers the synthesis of pro-inflammatory cytokines, such as type I IFN. Furthermore, this signal is crucial for activating DCs and facilitating antigen presentation to T cells [7,32,42]. Next markers principally associated with ICD are HSP70 and HSP90. These chaperones, present in the cytoplasmic compartment, are involved in protein folding and can be upregulated to express protective response to stress conditions, such as heat shock. Moreover, both HSPs may be exposed on the cell membrane and act as “eat me” particles, attracting phagocytes and natural killer (NK) cells [30]. Moreover, HSP70 and HSP90 play important role in the cross-presentation of tumor-derived antigenic peptides on MHC class I molecules, providing the cytotoxic T lymphocytes (CTLs, CD8^+^) response [7].

**Figure 1:**
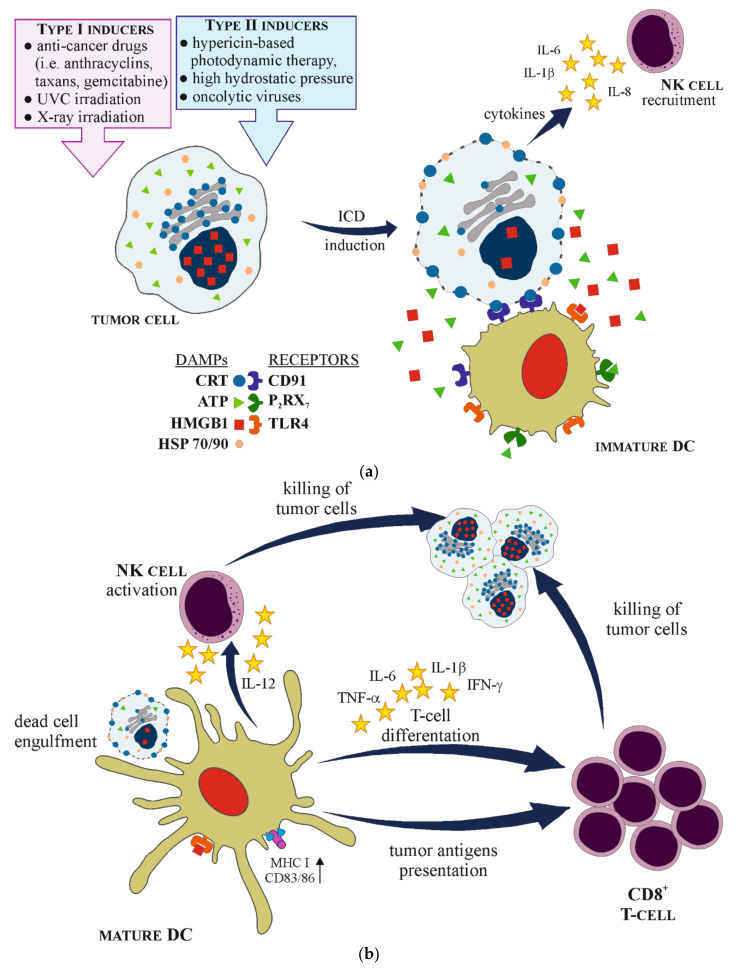
Schematic presentation of immunogenic cell death (ICD) mechanism. (**a**) ICD is induced after the exposure of tumor cell to type I or type II ICD inducers. Dying tumor cell releases and exposes on its plasma membrane different damage associated molecular patterns (DAPMs) molecules that are recognized by specific pattern recognition receptors (PRRs) on immature dendritic cells (DCs), leading to their maturation and activation. Additionally, tumor cells secrete cytokines that recruit natural killer (NK) cells. (**b**) Mature DC engulfs dying tumor cell, processes tumor antigens and presents it along with major histocompatibility complex (MHC) class I and costimulatory molecules (CD83/86) to CD8^+^ T-cells. Moreover, mature DCs secrete a range of cytokines that promote T-cells differentiation into CD8^+^ phenotype, as well as activate NK cells. Finally, activated NK cells and CD8^+^ T-cells are able to kill tumor cells.

The development of anti-tumor immune response, initiated by ICD, involves a few important steps: (1) induction of ICD by the exposure of tumor cell to specific ICD inducers; (2) changes inside the tumor cell, leading to ER stress; (3) chronic release and membrane exposure of DAMPs, especially CRT, HMGB1, HSP70, HSP90 and ATP; (4) recognition of DAMPs by particular PRR on APCs, mainly on DCs; (5) activation and maturation of DCs; (6) promotion of dying tumor cell engulfment by mature DCs; (7) processing of tumor-derived antigen specific cargoes inside DCs; (8) triggering T-cell immune response by tumor antigen presentation along with MHC I and costimulatory molecules, especially to CTLs; (9) killing of tumor cells. Each step can be amplified by the action of specific DAMPs [22,35,43]. Moreover, during and after the induction of ICD in malignant cells, numerous cytokines and chemokines are identified, for example pro-inflammatory cytokines such as IL-6, IL-1β or tumor necrosis factor α (TNF-α), which can increase the MHC class I expression on APCs, promote T cell differentiation and NK cell activation. Furthermore, activated DCs, release IL-12 that enhances the functionality of NK cells. On the other hand, tumor cells treated with ICD inducers also secrete immunomodulatory cytokines as IL-8 or IL-6 [30] (Figure 1).

## 3. Chaperones—Essential Molecular Regulators of Cell’s Proteins in Normal and Stress Condition

Chaperones are cellular proteins, which main function is to assist folding, assembly and guard proper conformational changes in molecules of numerous client proteins. This makes them very important modulators of many crucial cellular processes, including biosynthesis, maturation and degradation of proteins and/or their intracellular transport. What is more, they also control functions of enzymes, receptors or even transcriptional factors in a conformational-dependent manner [44,45]. Moreover, chaperones act as a stress-response, cytoprotective mechanism and contribute to cell’s quick adaptation to various stress-inducing agents, as well as ensure cell’s survival and recovery in pathological state. However, abnormal expression of molecular chaperones is also very often regarded as a biomarker of multiple diseases, including cancer [46].

All chaperones are divided into five distinct superfamilies: (1) heat shock proteins; (2) glucose-regulated proteins (GRPs); (3) Ca^2+^—binding chaperones; (4) protein disulfide isomerases; (5) peptidyl-prolyl isomerases [46,47]. Heat shock proteins compose majority of chaperones and are divided into six subfamilies, based on their molecular weight and structural homology: HSP110 (HSPH), HSP90 (HSPC), HSP70 (HSPA), chaperonins (including HSP60—HSPD and HSP10—HSPE), HSP40 (DNAJ), sHSPs (HSPB). HSPs reside mainly in cytosol but some (such as TRAP1) are also located in mitochondria [48,49,50]. Glucose-regulated proteins are another superfamily of chaperones, which involves four subfamilies: GRP170, GRP94, GRP78 and GRP75. Members of these families can be located in cytosol, nucleus, mitochondria, on the cell surface or even secreted into extracellular milieu [51]. Ca^2+^ binding chaperones include two members: calreticulin and calnexin, both of which are preferentially localized in ER and also on the cell surface [52,53]. The fourth chaperones superfamily consists of protein disulfide isomerases, which generally act as thiol-disulfide oxidoreductases and disulfide isomerases. These enzymes either form or break disulfide bonds between cysteine residues in proteins, thus affecting their folding and stability. Members of this superfamily are present in cytosol, nucleus, mitochondria, on the cell surface and are secreted extracellularly [54]. The last superfamily of chaperones are immunophilins, which are ubiquitous peptidyl-prolyl isomerases. These enzymes bend polypeptide chains at the sites of L-proline location and generally act as co-chaperones in the cooperation with HSP90/HSP70 [55].

Most of chaperones are constitutively expressed in normal conditions and exert their functions in unstressed cells. They catalyze proper folding, maturation (or degradation) of client proteins, as well as their transport within cell. Moreover, they regulate intracellular signal transduction and redox homeostasis. Some of these molecules (i.e., GRP170, GRP94, CRT), which are exposed on the cell surface or secreted into extracellular microenvironment promote cells exposition and secretion of particular peptides [46,56]. Under the condition of proteotoxic stress, (caused by heat shock, hypoxia, hypoglycemia, energy depravation, low pH, ROS generation or many other factors), cellular proteins undergo conformational changes and have a tendency to aggregate with each other. The inducible forms of chaperones bond to these stress-damaged proteins and ensure their proper folding and renaturation or, if this is impossible, initiate their degradation. Prevention of protein aggregation in cytosol, ER and mitochondria is the main chaperon-mediated mechanism protecting cells from death/being damaged [47,57].

## 4. Calreticulin—An Important, Multifunctional Player in Cell Biology

### 4.1. Structure

Calreticulin is a Ca^2+^ binding protein, coded by *CALR* gene located on chromosome 19p13.2. It is present in all cells of higher organisms, has a molecular weight of 46-kDa and consists of 417 amino acids with highly conserved sequence among diverse species [58,59]. This chaperone contains three distinct structural and functional domains: N-globular domain, P-arm domain and C-domain. N-terminal region of CRT is a lectin-like globular domain, containing eight antiparallel β-strands, oligosaccharide- and polypeptide-binding sites, as well as cleavable signal sequence. It is responsible for this chaperone interactions with DNA, α-integrins and binding of Zn^2+^ ions [15,60,61]. The proline-rich P-domain is located in the middle of calreticulin’s amino acid sequence and contains two sets of three repetitive regions [62]. These repeated sequences are believed to be involved in oligosaccharide binding together with N-terminal domain and form lectin-like structures responsible for protein-folding ability of CRT. Moreover, P-domain has also been found to bind Ca^2+^ with high affinity (K_d_ = 1 µM) and low capacity (1 mol of Ca^2+^ per 1 mol of protein) [15,63]. The C-terminal domain of CRT is a highly acidic region, composed mainly of negatively charged residues, which are interrupted with basic K or R residues (at regular intervals). This domain is followed by KDEL sequence responsible for chaperone retrieval from Golgi apparatus to endoplasmic reticulum [14]. C-domain is important for its Ca^2+^ buffering functions. In contradiction to P-domain, it can bind Ca^2+^ with low affinity (K_d_ = 2 M) and high capacity (25 mol of Ca^2+^ per 1 mol of protein) and is known for binding almost 50% of Ca^2+^ amount within ER [15,64].

### 4.2. Localization and Functions

**Figure 2:**
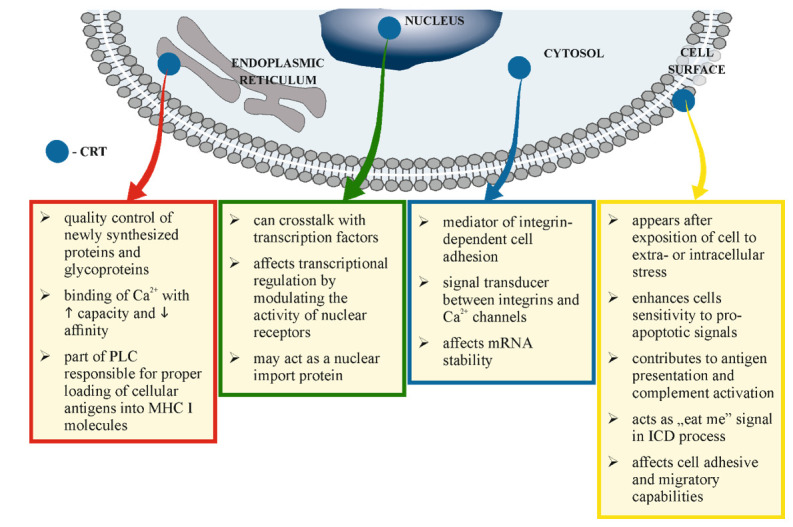
Multiple functions of calreticulin depending on its cell’s localization.

Since CRT contains cleavable signal sequence (N-domain) and KDEL ER retrieval sequence (C-domain), it is not surprising that this protein is predominantly present in endoplasmic reticulum (including nuclear envelope and smooth ER). However, this chaperone has also been found in cytosol, nucleus or even on the cell surface [65,66,67]. Depending on its cellular localization, CRT may manifest various biological functions within the cell Figure 2.

#### 4.2.1. Endoplasmic Reticulum

CRT located in ER has two major functions: protein chaperoning and regulation of Ca^2+^ homeostasis. This lectin-like chaperon is involved in the quality control system for newly synthesized proteins and glycoproteins, such as integrins, surface receptors or transporters, and supports their refolding, thus preventing premature export of misfolded proteins from ER. It is worth to mention that apart of calreticulin, this system relies also on additional chaperones (i.e., calnexin (CANX), GRP94, GRP78 or ERp57) and is commonly known as CALR/CANX cycle [68,69,70]. Moreover, CRT is an integral part of peptide-loading complex (PLC), responsible for proper loading of cellular antigens into MHC class I molecules [16]. This multicomponent complex, involve other proteins, such as PDIA3, TAP-binding protein or ATP-binding cassette subfamily B member (TAP1 and TAP2). They collectively mediate the following cascade of events: (1) assembly of MHC class I heavy chains with beta 2 microglobulin; (2) transportation of cytosolic proteins into ER lumen and their loading on antigen pocket of MHC class I molecules; (3) release of loaded molecules for anterograde ER-Golgi transport; (4) exposure of MHC class I molecules on the plasma membrane [71]. CRT contributes to PLC by preserving steady-states MHC class I heavy chains, as well as retrieving sub-optimally assembled MHC class I molecules from post-ER compartments (mainly ER-Golgi intermediate compartment) [72,73].

Another central function of CRT is binding calcium ions with high capacity and low affinity. This ability makes it a class I Ca^2+^ binding protein and a major ER buffer [14]. Since Ca^2+^ is mainly accumulated in ER, the expression of calreticulin affects its storage capacity [74]. It has been shown that overexpression of CRT may lead to increased intracellular Ca^2+^ storage, whereas CRT-deficient cells have lower capacity to harbor these ions [14,75]. This action of calreticulin has a very important functional implications, since Ca^2+^ is essential for many ER activities, such as protein folding, lipids and steroids synthesis, post-translational modifications, or activation of various transcriptional cascades [58]. From the point of view of cell functioning, CRT-dependent regulation of calcium concentration in the ER results in induction of Ca^2+^-dependent signaling pathways, integrin signaling, cell’s response to apoptosis or regulation of Ca^2+^-activated perforin and granzymes/proteases release by cytotoxic T-cells upon interaction with target cells [15,58,76].

#### 4.2.2. Cytoplasm

The process of calreticulin translocation to the cytoplasm is still not well understood. Generally there are various mechanisms proposed as the cause of this chaperone cytoplasmic localization: (1) CRT is not appropriately targeted for the transport to ER and thus its precursor is accumulated in cytoplasm; (2) CRT leaks out of the ER into cytoplasm; (3) CRT is being retrieved from ER by reverse movement after removal of its signaling peptide sequence; (4) CRT is being released into cytoplasm due to Ca^2+^ depletion in ER [15,77]. Cytoplasmic calreticulin is considered to be integrin activator and mediate integrin-dependent cell adhesion by direct interaction with cytoplasmic tail of α-integrin through KXGFFFKR sequence. Moreover, CRT acts as a signal transducer between integrins and Ca^2+^ channels in the plasma membrane [15,78]. It has been also described that CRT can interact with mRNA and significantly affect its stability. This mechanism of post-transcriptional processing of cytosolic mRNA is based on CRT binding to AU-rich region in 3′-UTR of angiotensin II type I (AT1) receptor mRNA, leading to its destabilization. This receptor is involved in regulation of cell growth, vasoconstriction or free radical release, however it has also been implicated in the development of cardiovascular disease [79]. Additionally, it was reported that CRT binds to 3′-UTR element of glucose transporter-1 (GLUT-1) and destabilize mRNA under high-glucose conditions in vascular endothelial and smooth muscle cells, what protects cells against excessive glucose influx an its deteriorating effects [80].

#### 4.2.3. Nucleus

There is very little known about calreticulin presence and functions in nuclear matrix. It has been suggested that CRT can crosstalk with transcription factors and affect transcriptional regulation by modulating the activity of nuclear receptors. This may be done either by its direct interaction with specific amino acid sequence (KXFFKR) or indirectly through Ca^2+^-dependent signaling pathways, such as Ca^2+^/calmodulin/calreticulin/NF-AT or Ca^2+^/Ras/MAPK pathway [58]. There are also reports indicating that CRT may act as a nuclear import protein [15].

#### 4.2.4. Cell Surface

**Figure 3:**
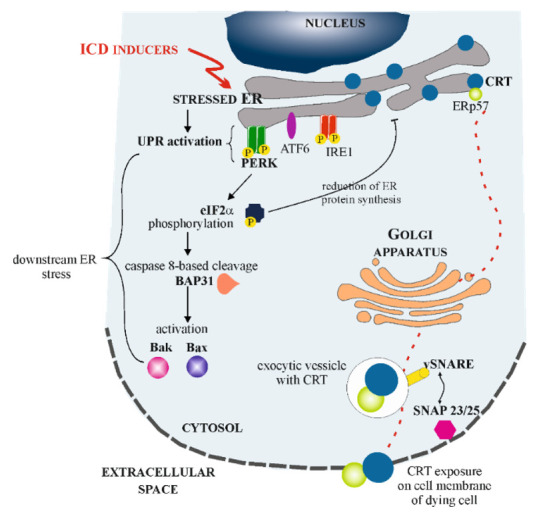
Mechanism of calreticulin (CRT) exposure in the response to immunogenic cell death (ICD) inducers. After exposition of cells to stress factors causing ICD, the loss of ER homeostasis leads to activation of unfolded protein response (UPR) signaling pathways. During ICD, protein kinase R-like endoplasmic reticulum kinase (PERK) activation leads to the phosphorylation of α-subunit of eukaryotic initiation factor 2 (eIF2α), inducing the downstream ER stress: pre-apoptotic, caspase 8-based cleavage of B-cell receptor-associated protein 31 (BAP31) and activation of BCL2-associated X protein (Bax), as well as Bcl-2 homologous antagonist/killer (Bak). Finally, CRT that has transited to the Golgi apparatus is exocytosed through soluble N-ethylmaleimide-sensitive fusion protein-attachment protein receptor (SNARE)—dependent pathway. Exocytic vesicles fuse with plasma membrane by interactions between SNAREs related with vesicles and synaptosome associated protein 23/25 (SNAP23/25) associated with cell membrane and CRT is exposed on the cell membrane of dying cell.

Since calreticulin possess KDEL sequence, under normal conditions, this protein can safely traffic proteins between ER Golgi complex, intermediate ER-Golgi complex, trans Golgi network or cytoplasm and it will always be retrieved to ER. Moreover, the physiological isoform of calreticulin cannot reach cell surface as it is non-glycosylated protein and thus cannot be exposed via anterograde type secretory pathways [81]. However, CRT may appear on the surface of cells exposed to various extracellular or intracellular stress factors (i.e., hypoxia, high temperature, Ca^2+^ or pH imbalance, cytokines or ICD inducers). Cells react to these agents by undergoing integrated stress response (ISR), what in majority of cases results in a loss of ER homeostasis. This in turn, leads to the activation of UPR. It is mediated by three stress sensors (as was described in chapter 2): IRE1, PERK and ATF6, all of which are maintained in inactive state under physiological conditions. During ICD, PERK plays crucial role. Its activation leads to phosphorylation of eIF2a, what reduces the amount of protein synthesis in ER. This leads to the induction of downstream ER stress: pre-apoptotic, caspase 8-based cleavage of B-cell receptor-associated protein 31 (BAP31) and activation of BCL2-associated X protein (Bax), as well as Bcl-2 homologous antagonist/killer (Bak). Simultaneously, CRT derived from ER is exocytosed through soluble *N*-ethylmaleimide-sensitive fusion protein-attachment protein receptor (SNARE)—dependent pathway. Exocytic vesicles, formed in Golgi apparatus, fuse with plasma membrane by interactions between SNAREs related with vesicles and synaptosome associated protein 23/25 (SNAP23/25) associated with cell membrane [17,82] (Figure 3). It is important to underline that calreticulin co-translocates to cell surface with ERp57, which tightly binds to the tip of calreticulin’s P-domain in ER lumen [83]. This member of protein disulfide isomerases is necessary for translocation process and it has been shown that disruption of CRT-ERp57 interaction stops calreticulin’s exposure on the cell surface [84,85].

**Figure 4:**
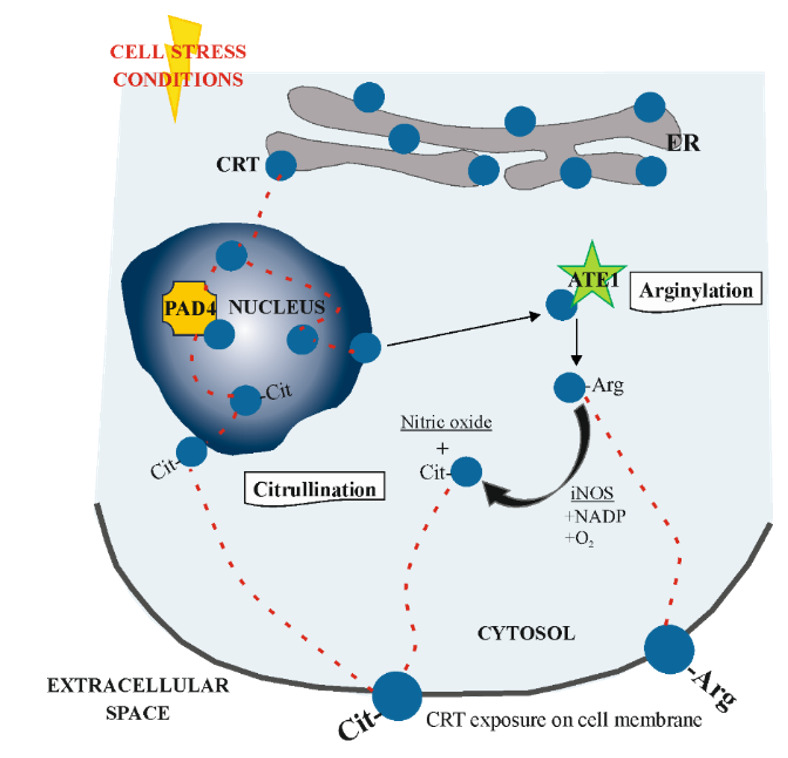
Mechanism of retrotranslocation of calreticulin (CRT) to cytoplasm. Under cell stress condition, CRT leaves the properly functioning ER and translocates to the nucleus. Here, it is exposed to protein arginase deaminase 4 (PAD4) and may be citrullinated (Cit-CRT), or not, before shuttling to the cytoplasm in association with nuclear export proteins. In cytosol CRT interacts with arginyl-tRNA transferase (ATE1), thus being arginylated (CRT-Arg). This isoform can be further citrullinated in the presence of inducible nitric oxide synthase (iNOS), as a byproduct of nitric oxide production from the conversion of arginine to citrulline. Both isoforms of CRT are identified in the plasma membrane of the cell.

Apart from UPR, there is some evidence that calreticulin may be retrotranslocated to cytoplasm from properly functioning ER, particularly when the cell is under stress condition, where it undergoes post-translational modifications [86]. The group of Afshar et al. [87] suggested that this process occurs via nucleus and is ubiquitin- and proteasome-independent. In nucleus CRT interacts with protein arginase deaminase 4 (PAD4), may be citrullinated (CRT-Cit) or not (CRT) and shuttled to the cytosol in association with nuclear export proteins. There, CRT interacts with arginyl-tRNA transferase (ATE1), which arginylates this protein (CRT-Arg). This isoform can be also citrullinated in cytosol in the presence of inducible nitric oxide synthase (iNOS), as a byproduct of nitric oxide production from the conversion of arginine to citrulline. Both citrullinated and arginylated isoforms of calreticulin have been found exposed on the cell membrane [81] (Figure 4).

Moreover, it has been indicated that in some cancer types, specifically myelofibrosis and thrombocythemia, calreticulin carries type I and type II mutations in exon 9. Mutated CRT has compromised KDEL domain, what enhances its extracellular secretion [88]. Therefore, it can be stated that surface CRT is generally observed only in abnormal cells (i.e., mutated or cancer cells) or cells that are affected by prolonged exposition to stress factors [89,90].

Cell surface calreticulin plays important role in cell survival and apoptosis by enhancing cell sensitivity to pro-apoptotic signals. It is involved in the immune system response as it contributes to antigen presentation and complement activation. Moreover, CRT exposed on the cell membrane may affect cell adhesive and migratory capabilities by interacting with thrombospondin 1 (THBS1), which participate in the regulation of integrin-independent cell adhesion [91]. More importantly though, ecto-CRT is considered to play essential role in the initiation of immunogenic cell death in pre-apoptotic cells [15,92].

### 4.3. Calreticulin in Danger Signaling—Implications in ICD

Calreticulin is probably the best identified DAMP and is also considered to be one of the main hallmarks of ICD [18,19]. CRT exposed on the cell surface acts as “eat me” molecule by delivering pro-phagocytic signals to APCs, such as DCs and their precursors, thus initiating immunostimulation along with the uptake of dying cells [93]. The mechanism of this action is based on CRT binding to CD91 receptor (LRP1) on the surface of DCs. This leads to their phenotypic maturation and activation, initiating engulfment of pre-apoptotic cells. Moreover, CRT is involved in promoting presentation of target-antigens to CTLs [94,95]. This function is based both on promoting the expression of co-stimulatory molecules (i.e., CD83, CD86) on DCs, as well as on inducing the production of specific cytokines. CRT has been shown to stimulate DCs to produce mainly NFk-β-dependent pro-inflammatory cytokines, such as IL-1β, IL-6 and TNF-α, leading to increased T-cells Th1 and Th17 polarization. This results in the establishment of mainly cellular, CTL-mediated immune response with potential to kill cells via an IFNγ-dependent mechanism. Immunogenic function of CRT is further substantiated by the fact that this protein does not induce production of anti-inflammatory IL-10 by DCs [96,97].

It is worth to mention that CRT-mediated immunogenic phagocytosis of dying cells can be inhibited by at least two different mechanisms: exposition of phosphatidylserine (PS) and CD47 receptor. PS is presented in high amounts on the surface of cells that undergo caspase-dependent apoptosis. Extracellular PS is recognized by macrophages with receptor jumonji domain-containing 6 arginine demethylase and lysine hydroxylase (JMJD6, also known as PSR). This allows macrophages to rapidly clear dying cells in an immunologically silent way, before CRT is even detected by APCs [98,99]. CD47 on the other hand acts as an anti-phagocytic, “do not eat me” signal and binds to signal regulatory protein alpha (SIRPA) on the surface of APCs, thus actively inhibiting phagocytosis [100]. As it turns out, the rapidity and relative level of CRT exposition determines the ultimate outcome of interaction between dying cells and phagocytes.

### 4.4. Mechanisms of Calreticulin-Dependent ICD in Tumor Cells

It has been found that surface exposure of CRT, is a condition sine qua non for the immunogenicity of dying or dead cancer cells and the induction of tumor-targeting immune response by cancer cells undergoing ICD [93]. However, it is important to underline, that although CRT exposition is required, it is not sufficient by itself for cell death to be considered as immunogenic [26]. As it was described above, the main mechanism of CRT pro-phagocytic action is based on its binding with CD91 on the surface of APCs and activating immature DCs. It leads to direct engulfment of dead or dying tumor cells by phagocytes, as well as tumor antigen-presentation to CTLs. Additionally, it has also been shown that mature DCs, enhance tumor lymphocyte infiltration and increase responsiveness to cancer immunotherapy in a CRT-dependent manner [35,101].

Interestingly, other mechanisms of CRT tumor-associated functions, which are not directly connected with phagocytosis of dying cells, have been also reported in acute myeloid leukemia (AML) [20]. High exposure of CRT in AML blasts has been shown to favor the accumulation of CD11b^+^CD14^+^ population of myeloid cells. They express IL-15 receptor alpha chain (IL15RA) and maturation markers, what allows them to present IL-15 to NK cells, thus enhancing their effector function against AML cells [102]. On the other hand, CRT exposed on the surface of AML cells has been described to be associated with type I interferon signaling. Instead of classical DC maturation markers, CRT induced expression of type I IFN, which in turn lead to IL-10 production by cancer cells and development of leukemia-specific T-cell immunity [103].

## 5. The Role of Calreticulin in Epithelial Ovarian Cancer

The ovarian cancer is the most-deadly gynecological malignancy. Due to the lack of characteristic symptoms and efficient screening tests, 70% of patients are diagnosed in an advanced stage of disease (III and IV stages at the FIGO classification), when cancer cells have already migrated from primary tumor. The 5-year-survival of women with advanced ovarian cancer is very low and does not exceed 25%. Based on an origin, the ovarian cancer is divided into three types: epithelial (95%), germ cells (3%) and sex-cord stromal (2%). According to the histological categorization five main subtypes of EOC are distinguished. The most common (70–80%) is a high-grade serous carcinoma, the rest are endometrioid, clear cell, mucinous and low-grade serous carcinomas [23,24,25]. Moreover, in regard to the molecular, genetic, clinical and histopathological studies all EOC are also classified as type I and type II. Type I tumors develop from well-established precursor lesions and are genetically stable. In this group low-grade serous, mucinous, endometrioid and clear cell carcinomas are included. In contrast, type II tumors characterize with lack of well-defined precursor lesions, high aggressiveness and genetic instability. Among them the high-grade serous carcinomas, as well as the undifferentiated carcinomas predominate [104,105,106].

An important mechanism by which ovarian cancer cells evade the immune response, is its poor immunogenic character. This problem can be overcome by better identification and evaluation of immune-modulating therapeutic approaches [107]. It is known that some kind of treatments cause apoptotic cell death, which is known as ICD, leading to the presentation of dying cancer cells to the immunocompetent cells and subsequent development of anti-cancer immune response [24]. Below we have summarized the involvement of CRT, known for its implication in the ICD, in the ovarian cancer cells treatment and on the ovarian cancer patients’ survival.

### 5.1. Studies Assessing the Ability of Anti-Ovarian Cancer Agents to Induce the Surface Expression of Calreticulin (ecto-CRT)

The treatment of EOC patients traditionally includes a combination of chemotherapy and cytoreductive surgery. Despite slight differences in the individual protocols of chemotherapy, the general strategy is common in the United States and the European countries. The first line of treatment consists of platinum compound—carboplatin and taxanes—paclitaxel and docetaxel. The drugs recommended for recurrent disease are carboplatin and taxanes enriched with other therapeutics such as gemcitabine, liposomal or standard doxorubicin, bevacizumab, topotecan, etoposide and others being under different phases of clinical studies. Unfortunately, the effectiveness of EOC’s cytotoxic treatment is not satisfactory and high resistance of ovarian cancer cells to platinum and taxanes is, next to late diagnosis, main reason of patients relapse and high rate of death [24,108].

The studies assessing the ability of drugs or physical factors to induce ICD are based on: (1) detection of DAMPs, primarily ecto-CRT, extracellular release of ATP and HMGB1, as well as extracellular expression of HSP70 and HSP90; (2) determination of immunological parameters strongly connected with ICD, i.e., expression of CD91 and CD14 molecules or TLR4 on the surface of APCs; (3) stimulation of type I IFN responses [34,36,43]. Since, this paper is dedicated to calreticulin only, below we have presented the review of available data on the capacity of anti-ovarian cancer drugs to the induction of ecto-CRT on ovarian cancer cells. If available the ICD-related consequences of calreticulin expression or other mechanisms influencing tumor cells have been also described. However, it should be underlined that available data on the drugs’ induction of CRT in ovarian cancer cells is highly limited (Table 1).

#### 5.1.1. Platinum Compounds

Cisplatin, carboplatin and oxaliplatin are main platinum derivatives used in clinic. They are alkylating agents, which principal action is based on the formation of intra- and inter-strand connection between DNA strands. However, platinum compounds exert also other effects i.e., they can increase intracellular amount of ROS influencing ER stress [37,117,118]. Despite the high controversy, many reports clearly indicate that cisplatin or its analogs can effectively induce the expression of ecto-CRT on ovarian cancer cells. It was described that human ovarian adenocarcinoma cell lines—SK-OV-3 cultured with cisplatin expressed membrane CRT. Moreover, sequential IFN-β and cisplatin treatment of SK-OV-3 cells enhanced surface level of CRT in comparison to drug alone. It indicates that pre-treatment of cancer cells with IFN-β makes them more immunogenic [109]. Other studies proved that exposure of 2F8 cells (cell line which was derived by authors from Cre-encoding adenovirus (AdCre)-induced orthotopic ovarian tumors) to cisplatin induced the ecto-CRT expression on these cells [110]. Yet another report showed that free oxaliplatin or phase-transition nanoparticles loaded with oxaliplatin caused translocation of CRT to the surface of ID8 mouse ovarian surface epithelial cell line, which is used as a syngeneic mouse model for human ovarian cancer. Moreover, studies demonstrated that the inhibition of ROS production in ID8 cells completely blocked appearance of surface CRT, pointing at ROS requirement in oxaliplatin-mediated chaperone expression [111].

The effectiveness of cisplatin to induce ecto-CRT was also proven in the case of other tumor cells. For example, cisplatin and carboplatin caused CRT translocation from ER to membrane surface of human lung adenocarcinoma cell line A549 [119]. It was also found that cisplatin and oxaliplatin increased cell surface levels of CRT and HSP70 on various human cancer cell lines: head and neck squamous [120], colon (Caco2), testicular (833KE and 2102EP) and melanoma (BLM) [121].

#### 5.1.2. Doxorubicin

Doxorubicin is an anthracyclines’ class of drug with the ability to the intercalate into DNA and disrupt topoisomerase-II-mediated DNA repair, as well as to induce free radicals generation [122]. The capacity of doxorubicin and other anthracyclines to upregulate surface CRT, although widely accepted, is not well documented in ovarian cancer model. One study demonstrated that doxorubicin successfully induced significant translocation of CRT to the surface of human ovarian cancer cell line OV90. However, what is more valuable, similar effect was noticed using the primary ovarian cancer cells that were isolated form tumors of ovarian cancer patients (*n* = 5), who underwent surgical operation [112]. Yet another study was aimed to test usefulness of CXCR4 antagonist-armed viral oncotherapy in the combination with doxorubicin to overcome ovarian cancer cells chemoresistance. In this research, paclitaxel and carboplatin resistant mouse ID8-R and human CAOV2-R ovarian cancer cells were used as a model. The viral treatment of these cell lines, followed by doxorubicin administration successfully induced surface exposure of CRT. Moreover, the appearance of “eat me” signal on cancer cells enhanced the effectiveness of DCs in phagocyting them [113]. In the case of other malignant cells, doxorubicin was also described to be effective for the induction of cell’s surface expression of CRT on GBM human glioblastoma cell line [123].

#### 5.1.3. Paclitaxel

Paclitaxel is a member of taxanes family, which activity is based on arresting mitosis through microtubule stabilization, leading to the apoptosis of cell [124]. Data published recently by Lau et al. [114] showed that paclitaxel (but not cisplatin and carboplatin) effectively induced membrane CRT expression on human SK-OV-3 and mouse ID8 ovarian cancer cell lines, in vitro. Moreover, studies evidenced that this drug activates TLR4-dependent signaling pathway in cancer cells, which is essential for cell surface expression of CRT. Comparable results, showing the ability of paclitaxel to induce CRT exposure, were presented on A549 human lung adenocarcinoma cell Line [119].

#### 5.1.4. Other Agents

High resistance of ovarian cancer patients to platinum compounds and taxanes forces to test new drugs, potentially useful in the therapy of this malignant tumor. One of the research pathways is using inhibitors of signaling proteins important for tumor cells’ grow, survival and invasion. The bromodomain and extraterminal (BET) family proteins have multiple functions and i.e., participate in the regulation of apoptosis and cell cycle. The benefit of using BETs inhibitors in a targeted therapy of various malignant tumors, including ovarian cancer, has been studied and described [125,126]. The ability of BETs inhibitors to induce ICD was also determined. Using human ovarian cancer cell line CAOV-3, Liu et al. [115] demonstrated that i-BET151 (BET inhibitor) promoted the translocation of CRT from ER to cell surface. Parallelly, this agent enhanced the number of apoptotic cells and inhibited the formation of clones.

Resveratrol (RES) is non-flavonoid polyphenol with documented anti-inflammatory, anti-microbial and anti-oxidant activities. It has been also tested for the anti-cancer properties [127]. However, the ability of RES to develop ICD in ovarian cancer is at the beginning of study. One report of Zhang et al., [116] showed that RES exposition to SK-OV-3 and A2780 human ovarian cancer cell lines, as well as murine ovarian carcinoma ID8 cells, resulted in appearance of membrane CRT. Moreover, the authors showed that RES effectively stimulated the infiltration of high level of DCs (CD80^+^/CD86^+^) and cytotoxic CD8^+^ T-cells into tumor tissue of xenograft tumor model established with ID8 cells.

### 5.2. Studies Assessing the Impact of CRT on Ovarian Cancer Cells Activity and Ovarian Cancer Patients Survival

Elevated level of ecto-CRT in numerous tumor cells (i.e., lung, colorectal, ovarian, acute myeloid leukemia) has been considered either as superior or inferior molecule enhancing the anti-tumor immunity. Moreover, the controversies around CRT’s prognostic and/or predictive value are substantiated by the fact that wide range of studies, involving patients suffering from various kind of tumors, brought up opposed results. In many cohort studies (i.e., non-small cell lung carcinoma, ovarian cancer, primary myelofibrosis) high CRT level indicated on improved overall survival of patients. In contrast, high level of CRT was also connected with negative prognostic value in, i.e., neuroblastoma, pancreatic cancer and mantle cell lymphoma [20,128]. Below we have presented the available data concerning the pros and cons of CRT in ovarian cancer model.

Interesting paper concerning correlation between CRT expression and infiltration of immune cells in tumor tissue of ovarian cancer patients was published by Stoll et al. [129]. The authors conducted the transcriptome analysis of human ovarian cancer tissue (*n* = 520) for mRNA level of CRT in correlation with metagenes connected with activated DCs (aDCs) and CTLs. The data clearly indicated that *CRT* expression affected the amount of infiltrate immune cells. High level of *CRT* mRNA positively correlated with higher presence of aDC and with higher level of aDCs and CTLs together. Moreover, the combination of CRT+aDCs or CTR+aDCs+CTLs had significant impact on the overall survival (OS) of ovarian cancer patients. This data evidenced that CRT positively affected patients’ survival. The observed effect was dependent on infiltration of tumor tissue with DCs and CTLs, both of which are principal players in the anti-tumor immune response.

Other studies based on the analysis of TCGA, EGA or GEO databases showed the beneficial impact of high *CRT* expression on OS and progression-free survival (PFS) of ovarian cancer patients. The research described by Garg et al. [130] included ovarian cancer patients treated with topotecan non-ICD inducer (*n* = 119) or with paclitaxel the inducer of ICD (*n* = 220). Patients were next divided into two groups based on either high or low *CRT* expression in cancer tissue (mRNA level). Data showed that paclitaxel-treated patients with high *CRT* expression demonstrated significantly better OS (HR = 0.54) and PFS (HR = 0.57) calculated up to 100 months, than patients with low *CRT* level but also undergoing treatment with paclitaxel. In contrast, *CRT* expression was not a determinant of topotecan-treated ovarian cancer patients’ survival.

Yet another article published by Kasikova et al. [131] also described the beneficial effect of CRT in ovarian cancer. The authors evidenced that this chaperone enhanced survival of patients with high-grade ovarian carcinoma and can be considered as a good prognostic factor. Study included immunohistochemistry staining of CRT in retrospective cohort of 152 patients, who did not receive any neoadjuvant therapy. It turned out that high expression of CRT significantly improved both OS and relapses-free survival (RFS) of women. Better OS in CRT^high^ group was especially noted in the case of advanced ovarian cancer (FIGO stage III/IV). The authors investigated also the possible mechanism involved in the translocation of CRT from ER to membrane in ovarian cancer cells. Thus, the mRNA level of 3 canonical factors inducing ER stress response in the context of *CTR* expression was tested. The significantly positive correlation between *CRT* and *DDIT3*, *HSPA5* and *HSP90B1* was found in both primary and metastatic tumor samples. Lastly, tumor specimens were tested for association between CRT expression and the type of cells infiltrating cancer tissue. Transcriptional and bioinformatics analysis demonstrated that in primary and metastatic tissue of high-grade ovarian cancer, elevated expression of CRT positively correlated with the expression of T-cells polarized into Th1 phenotype. It evidenced that CRT is responsible for induction of acquired immune response, which is required for successful eradication of cancer cells. Recent report also proved CRT to be a good prognostic factor in EOC patients. In the study 124 patients who underwent primary debulking surgery was enrolled. The ovarian tumor specimens were examined by immunochemistry method for CRT expression. The data showed that patients with higher CRT level had better OS and disease free survival rate [114].

A very interesting paper supporting positive, anti-tumoral impact of CRT on ovarian cancer cells was recently published by Schcolnik-Cabrera et al. [132]. The authors demonstrated that rTsCRT, a recombinant CRT obtained from parasite (*Taenia solium*), significantly reduced cell viability of SK-OV-3 ovarian cancer cell line, but not cell lines of other cancers such as breast—MDA-MB-231 or prostate—PC3. Moreover, rTsCRT sensitized SK-OV-3 cells to 5-FU. Ovarian cancer cells, resistant to this drug, were treated with combination of rTsCRT and 5-FU and showed significant drop in viability and clonogenic potential.

However, there are also report showing lack of correlation between CRT expression and patients’ survival. Vaksman et al. [133] tested samples of 56 tumors classified as high-grade serous carcinoma (33 effusions, 14 primary carcinomas, 9 solid metastasis) for CRT expression on both mRNA (RT-PCR) and protein (western blot) levels. 41 patients enrolled in the study had primary surgery, while 9 secondary surgery and only 4 women received platinum-based therapy. The mRNA and protein levels of CRT were significantly elevated in solid metastases with very low level in effusions and primary tumors. In general, lack of any positive effect of CRT expression on OS and PFS of patients was observed. The one exception was an observation that high protein level of CRT positively correlated with complete response to chemotherapy at diagnosis.

In contradiction to well-described beneficial role of high CRT expression on EOC patients’ survival, its negative, pro-tumoral impact on ovarian cancer cells has also been reported. For example, it was noted that this chaperone can facilitate cancer cells migration and survival, in vitro. Silencing CRT (with shRNA) in SK-OV-3 ovarian cancer cell line resulted in seven-fold reduction of their viability during culture in standard medium. Moreover, cells have lost their ability to proliferate. It indicate that CRT can control the growth and survival of ovarian cancer cells [134].

Another study was focused on evaluation of CRT expression (mRNA, RT-PCR and protein levels, Western blot) in samples obtained from EOC, benign ovarian tumors and borderline ovarian tumors [135]. The data showed that CRT levels were significantly increased in tissue of EOC patients in comparison to benign and borderline tumors. It can point out on CRT participation in ovarian cancer development. This hypothesis can be supported by the fact that normal ovarian tissue characterized with very low or undetectable level of CRT. Moreover, authors focused on the potential factor responsible for initiation of CRT exposure in ovarian cancer tissue. The in vitro research on human ovarian cancer cell line A2780 showed that nerve growth factor (NGF) effectively stimulated cells for CRT expression. It should be underlined that NGF is overexpressed in tumor microenvironment of EOC [136,137]. More recent studies by Vera et al. [138] confirmed involvement of NGF in the induction of CRT translocation from ER to plasma membrane of A2870 cells.

## 6. Conclusions

The importance of CRT in the maintenance of cell homeostasis is well established and does not raise any doubts. Among the best recognized activities of this chaperone are: regulation of Ca^2+^ homeostasis; quality control for newly synthesized proteins and glycoproteins; participation in MHC class I antigen presentation or facilitation of cell adhesion. However, CRT is also the best recognized DAMP molecule and plays a key role in the initiation of immunogenic cell death. CRT present on the membrane of dying cells serves as “eat me” signal to APCs, such DCs and their precursors, as well as initiates immunostimulation. CRT and ICD have been best recognized in the context of therapy of malignant diseases, since both chemotherapeutics and photodynamic therapy are strong inducers of immunogenic cell death. Surface exposure of CRT is believed to help immune cells to kill tumor cells and enhances their poor immunogenicity. With late diagnosis and poor prognosis, ovarian cancer remains one of the most-deadly gynecologic malignant diseases of women. However, improving standard therapy either by finding efficient prognostic factors or by implementing immune-modulating therapeutic approaches can increase patients’ chances of survival. Nevertheless, the studies describing the impact of standard chemotherapeutics, used in the treatment of ovarian cancer, on the induction of surface CRT and ICD are merely beginning to develop. While, expression of CRT in ovarian tumor tissue has been found to be a useful indicator of patients’ survival, there is still a lot of controversies around its role, since CRT has been proven to work both ways, facilitating either better or worse patients’ OS. So far it is not possible to ultimately conclude whether this chaperone can be a promising indicator of patients’ survival, as up to this date available data on CRT expression in ovarian cancer is strongly limited.

## Figures and Tables

**Table 1:** The anti-ovarian cancer agents as an inductor of calreticulin (CRT) on ovarian cancer cells.

Cells	Agent	Ref.
SK-OV-3 cell line	cisplatin cisplatin + IFN-β	[109]
2F8 cell line	cisplatin	[110]
ID8 cells	free oxaliplatin phase-transition nanoparticles loaded with oxaliplatin	[111]
OV90 cell line primary ovarian cancer cells isolated from patients tumor (*n* = 5)	doxorubicin	[112]
ID8-R (paclitaxel and carboplatin resistant) cell line CAOV-2-R R (paclitaxel and carboplatin resistant) cell line	CXCR4 antagonist-armed viral oncotherapy in the combination with doxorubicin	[113]
SKOV3 cell line ID8 cell line	paclitaxel	[114]
CAOV-3 cell line	i-BET151 (BET inhibitor)	[115]
SKOV3 cell line A2780 cell line ID8 cell line	resveratrol	[116]

## Data Availability

Not applicable.
